# The effect of temporal leadership on quiet quitting among primary healthcare workers: the chain-mediating role of time management competency and work-family enrichment and moderating role of organizational communication

**DOI:** 10.3389/fpsyg.2025.1616354

**Published:** 2025-09-03

**Authors:** Qianqian Xu, Zhikai Yu, Si Fan, Yuanyang Wu, Yanting Wang, Dongdong Zou, Jinwen Hu, Xinping Zhang

**Affiliations:** ^1^School of Medicine and Health Management, Tongji Medical College, Huazhong University of Science and Technology, Wuhan, Hubei, China; ^2^School of Chinese Language and Literature, Hubei University, Wuhan, Hubei, China

**Keywords:** quiet quitting, temporal leadership, time management, work-family enrichment, organizational communication, primary healthcare workers

## Abstract

**Introduction:**

Quiet quitting among primary healthcare (PHC) workers is prevalent and seriously reducing healthcare productivity and quality. Temporal leadership takes advantage of effective time scheduling, which can avoid unreasonable working arrangements and then mitigate quiet quitting. However, there is little known about the influence and mechanism of temporal leadership on quiet quitting. This study aims to explore the influencing mechanism of temporal leadership on quiet quitting among PHC workers, in addition to test the chain-mediating roles of time management competency and work-family enrichment of PHC workers, as well as the moderating roles of organizational communication.

**Methods:**

An on-site survey of 520 PHC workers was conducted in a health reform area. The participants were asked to complete five self-report questionnaires, including Temporal Leadership Scale (TLS), Time Management Scale (TMS), Work-Family Enrichment Scale (WFES), Quiet Quitting Scale (QQS), and organizational Culture Scale (OCS).

**Results:**

Temporal leadership, time management, and work-family enrichment all significantly and negatively predict quiet quitting among PHC workers. Time management and work-family enrichment played the chain-mediating roles between temporal leadership and quiet quitting. The relationship between temporal leadership and time management was moderated by organizational communication. Temporal leadership can mitigate quiet quitting among PHC workers by enhancing their time management competencies and work-family enrichment, and high levels of organizational communication can strengthen the effect of temporal leadership on time management competency.

**Conclusions:**

These findings highlight the importance of temporal leadership in health systems and provide an evidence-based strategy for leaders to effectively address quiet quitting.

## 1 Introduction

In the modern employment landscape, turnover remains a persistent challenge for organizations globally. Amidst the more noticeable resignations, a subtler form of departure known as “quiet quitting” has emerged. The trend of quiet quitting is a new term to describe an increasingly common alternative to a great resignation. Quiet quitting refers to fulfilling only the essential duties of one's job without investing additional time, effort, or enthusiasm beyond what is strictly required, which nature is a kind of spiritual turnover behavior with concealment (Forrester, 2023). Contrary to what the name implies, quiet quitting does not involve the worker actually resigning from their position; instead, they continue in their position while receiving compensation but limit their efforts in work (Patel et al., 2025). While burnout among employee is well-documented, characterized by emotional exhaustion, depersonalization, and loss of professional fulfillment, and linked to depression, anxiety, suicidality, and negative patient outcomes. In contrast, “quiet quitting,” often seen as a subtle form of burnout, remains less understood (Chen and Meier, 2021). The quiet quitting phenomenon is becoming popular and has attacked global workforces in many industries, which attracts worldwide attention (Xueyun et al., 2023). According to the Gallup Report, the percentage of quiet quitters in the US workforce is close to 50%. Additionally, the ratio of employee engagement to actively separated employees fell from 2.5:1 to 1.8:1, marking the lowest level of employee proactively involvement in the previous 10 years (Formica and Sfodera, 2022). The similar phenomenon is also occurring in medical system and even worse (Gordon et al., 2014; Kumar, 2023). For example, national doctors' strike in United Kingdom took place in 2023 (Sim et al., 2021). What's more, a quantitative study found that nearly 57.9% of primary healthcare (PHC) workers in Greece were quiet quitters (Galanis et al., 2024). PHC workers such as general practitioners play the important role in safeguarding health rights of the citizens (Assefa et al., 2018). A large number of quiet quitters among them leads to a decline in healthcare quality and productivity (Wallace et al., 2009), then even worse may threaten patients' life.

The reason why quiet quitting phenomenon occurs and becomes increasing serious across different industries have been explored a lot. As for the triggers of quiet quitting, there is a review have identified a variety of contributing factors, such as leadership style, work-family relationships, individual traits, organizational communication, and so on (Geng et al., 2025). Some qualitative and quantitative researches have also illustrated how these factors influence quitting related behavior, one of which emphasizes the growing importance of leadership style development, such as humble leadership, is a means of mitigating these effects (Zhu et al., 2019). This kind of positive leadership style fosters a positive work environment, enhances employees' sense of belonging and psychological safety, and thereby reduces the tendency for employees to quiet quitting. Work-family affairs including such as work-family balance, work-family enrichment, and work-family conflict all have been found effectively contributes to quiet quitting (Geng et al., 2025). Among these constructs, work-family enrichment, which emphasizes the mutual gains between work and family roles, has received relatively limited empirical attention compared to the more extensively studied concepts of balance and conflict (Gao et al., 2025). Nevertheless, recent research has begun to recognize its significance as a positive resource-based mechanism that may enhance employee engagement and mitigate related quitting outcomes like turnover intention (Chen et al., 2018). Time management competency as an individual trait, has been proposed a potential trait to help employees to manage their working time experience less stress and turnover (Aeon et al., 2021). Meta-analytic research has found that organizations had better to create a positive work environment, ensure open communication channels, and provide support and development opportunities for employees to prevent quiet quitting (Geng et al., 2025). Another qualitative research has also declared that communication connects employees closely with the organization and sustainably strengthens their work capabilities and commitment to the organization (Urbancová et al., 2024).

Despite emerging recognition of quiet quitting's antecedent triggers, the mechanisms and conditions under which these effects unfold remain underexplored. Existing theories of organizational behaviors including trait activation theory (Luria et al., 2019) and resource scarcity theory (Mehta and Zhu, 2016), highlight that the leadership style of a supervisor or manager plays a crucial role in managing subordinates' behaviors, such as work engagement, personal competencies and even aspects of their family lives (Hoonsopon and Puriwat, 2021; Hudon et al., 2024; Iida et al., 2024). We argue that that employees' time management competency and work-family enrichment, as a trait activated by leaders and a personal resource that helps individuals better manage the demands of both work and family roles respectively, may mediate the relationship between temporal leadership and their work outcomes.

Furthermore, although prior research has called for more in-depth investigation into how to mitigate quiet quitting phenomenon among PHC workers (Galanis et al., 2024), few studies have examined how leadership styles may buffer or exacerbate its effects. In medical context, existing study indicates that unreasonable work arrangements, such as intensive shift work, are major causes of quiet quitting related behaviors (Chen et al., 2024; Galanis et al., 2024; Yuan et al., 2024). Aiming to narrowing this gap, our study aims to explore the mechanism of temporal leadership, which is excellent at working time arrangements for employee's, on quiet quitting, aiming to provide effective management strategies for mitigating quiet quitting in health system.

**Figure 1:**
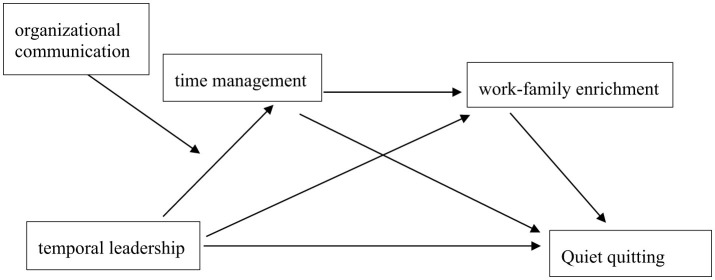
The theoretical model of the study.

Our study makes several primary contributions. First, it contributes to the literature about how to mitigate quiet quitting by introducing temporal leadership as a key antecedent within the context of primary healthcare, a field where time pressure and burnout are particularly prominent. Unlike traditional leadership styles, temporal leadership emphasizes the efficient use of time and coordination of work schedules, which may offer unique advantages in managing PHC workers' quitting behaviors. Second, this study develops and empirically tests a chain mediation model to explain how temporal leadership influences quiet quitting through two mediators: time management competency, based on trait activation theory, and work-family enrichment, grounded in resource conservation and trait activation theory. This study advances theoretical insights into the mechanisms linking leadership and quiet quitting by developing an integrated model grounded in trait activation theory and conservation of resources theory. In addition, the inclusion of organizational communication as a moderating variable enriches the contextual understanding of when temporal leadership is most effective, highlighting the importance of cultivating a transparent and supportive working environment. Furthermore, by focusing on primary healthcare workers, the study addresses the urgent need for context-specific research in healthcare management and offers practical strategies for enhancing workforce engagement and retention in frontline medical settings. Our integrated model is illustrated in Figure 1.

## 2 Literature review and hypotheses development

### 2.1 Temporal leadership and quiet quitting

Temporal leadership involves a particular type of leaders' time management behavior, in which leaders aim to help employees achieve efficient use of their time in carrying out their job duties (Mohammed and Nadkarni, 2011). It is a relatively new concept in the field of leadership studies that integrates two essential components: time and leadership (Ancona et al., 2001). Although it should be especially relevant to employees' job performance in time-constrained working environments, like medical workplaces, it is a new concept hasn't received much attention so far (Zhang et al., 2020). From a meta-analytic review of exploring triggers of quiet quitting, many findings underscore the importance of strong leadership, in fostering employee retention and engagement across various industries, including in medical work placement (Geng et al., 2025). Subgroup analyses in this review indicated that these supportive leadership styles which can support positive working environment can help reduce the likelihood of quiet quitting. As already mentioned above, unreasonable working arrangements are one of the primary risk factors for PHC workers to quiet quitting. Strong temporal leadership may influence the team's work performance through support a positive working environment with effective time scheduling and pacing, potentially helping to prevent unreasonable work arrangements and mitigate quiet quitting. This study will test following hypothesis:

H1. Temporal leadership is negatively associated with quiet quitting among PHC workers.

### 2.2 The potential mediating role of time management competency

Time management is defined as the ability that individuals use to construct, protect, and adapt their own time (Aeon and Aguinis, 2017). The primary distinction between time management and temporal leadership is that time management focuses on managing individuals' own time, whereas temporal leadership coordinates and manages the time of organizational members. Trait activation theory explored by several qualitative researches reveal that leader's temporal style is regarded as an important variable that influences an individual's temporal traits (Hoonsopon and Puriwat, 2021; Luria et al., 2019). Previous studies have argued that leadership should managers need to pay more attention to time management because their strong ability in managing time has a positive effect on their subordinates' organizational competencies, and finally reflecting the whole organizational time management competencies (Liu and Hallinger, 2018; Wang et al., 2011). To explore whether this effect still holds true for primary healthcare settings, our study assumes that temporal leadership can improve time management competencies of PHC workers.

It is generally considered that having good time management competency adversely predicts a number of undesirable behaviors. In education field, there are a great deal of researches exploring the effect of time management competency on learning behavior for students (Macan et al., 1990). Specifically, academic procrastination is thought to be a direct result of students' lack of time management competency, according to some academics (Liu et al., 2022). Additionally, a quantitative study indicates that time management inclinations have been shown to negatively influence procrastinating behavior for students (Liu et al., 2022; Trueman and Hartley, 1996). Similarly in health system, an extensive literature describes time management is a potential solution to reduce physician burnout (Nagle et al., 2024; Vinnikov et al., 2019; West et al., 2016), which also reduce high turnover rate (Aiken et al., 2023). These findings, although drawn from different domains, suggest a broader relevance of time management competencies in shaping professional behavior and well-being. Despite limited quantitative research on how time management affects emerging behavioral patterns such as quiet quitting in primary healthcare settings, it is theoretically plausible that similar associations apply. Specifically, inadequate time management may lead to increased stress, disengagement, and quitting related behaviors. So, our study assumes that time management competencies of PHC workers can mitigate their quiet quitting.

In view of above researches on the possible close relationship between temporal leadership, time management and quiet quitting, this study will test following hypothesis:

H2. Time management plays a mediating role in the relationship between temporal leadership and quiet quitting among PHC workers.

### 2.3 The potential mediating role of work-family enrichment

The concept of work–family enrichment is that experiences at work can improve the individual's satisfaction and role performance in family area (Greenhaus and Powell, 2006). Work–family enrichment can be improved by positive leadership styles (Braun and Nieberle, 2017; Zhang et al., 2012). Positive leadership is conceptualized as the application of a variety of positive practices that assist individuals and organizations in accomplishing goals, succeeding at work and home, feeling elevated vitality, and achieving levels of effectiveness (Azila-Gbettor et al., 2024). Positive leadership is not a distinct leadership concept, but rather an umbrella construct encompassing several leadership behaviors, including transformational, authentic, servant, ethical, etc. leadership styles (Hammond et al., 2015). Previous studies have shown that various positive leadership styles, including transformational leadership, significantly contribute to work–family enrichment (Wu et al., 2020; Zhang et al., 2012). Temporal leadership, as another form of positive leadership, may similarly support employees in achieving highly efficient use of time at work. This could suggest that temporal leadership might also play a role in enhancing work–family enrichment, though the specific mechanisms remain to be further explored.

The relationship between work-family enrichment and working behavior has been explored a lot. A previous study conducted 2-wave survey and revealed that work-family enrichment could improve employee's work performance like working engagement (Qing and Zhou, 2017). Therefore, the PHC workers' quiet quitting, the emerging negative behavior in workplace, may also be negatively influenced by work-family enrichment. Moreover, many previous researches indicated that work-family enrichment played a mediating role in the relationship between positive leadership such as supportive leadership, and occupational health such as individual's thriving at work (Russo et al., 2018). Based on this, this study will test following hypothesis:

H3. Work-family enrichment plays a mediating role in the relationship between temporal leadership and quiet quitting among PHC workers.

### 2.4 The potential chain mediating role of time management and work-family enrichment

According to the theory of resource scarcity, time is a finite resource and both work and family expect individuals to spend more time in their respective fields (Gilmore, 1974). Spending more time at work leads to less time to be devoted to the family area, resulting in lower level of work-family enrichment. Quantitative studies have shown that working hours are negatively correlated with employees' work-family balance, reducing the level of work-family enrichment (Wayne et al., 2007). Strong time management competency helps prevent procrastination, enables employees to complete tasks more efficiently, reduces working hours, and ultimately contributes to improved work-family enrichment. Based on this, this study will test following hypothesis:

H4. Time management competencies can positively predict work-family enrichment among PHC workers.

### 2.5 Moderating role of organizational communication

Organizational communication is defined as the exchange and transfer of information between members in an organization (Snyder and Morris, 1984). Conventional leadership study places a strong emphasis on the formal leaders‘ top-down influence over subordinates within a team or organization. The effectiveness of this influence is largely dependent upon organizational communication (Bolden, 2011). A quantitative study has indicated that organizational communication can effectively moderate the impact of abusive leadership on employee creativity (Wirawan et al., 2024). The relationships between employee time management competencies, temporal leadership and organizational communication have not received much emphasis in previous researches. This study attempts to investigate the moderating role of organizational communication between temporal leadership and employee's time management ability. High levels of organizational communication facilitate leaders with temporal leadership in effectively conveying work schedules to staff members, while also providing opportunities for employees to learn from leaders' positive attributes, such as time management competency. Based on this, this study will test following hypothesis:

H5. Organizational communication moderates the association between temporal leadership and time management competencies among PHC workers.

## 3 Methods

### 3.1 Participants and procedure

This is a cross-sectional study and PHC workers were surveyed on-site in all 11 primary healthcare institutions in a demonstrate county for poverty eradication of Hubei Province (Central China) in January 2024, whose management capacity including leadership has been significantly strengthened in the context of health poverty revitalization. The inclusion criteria were as follows: (1) general practitioners, nurses, medical technicians, public health physicians and other medical assistants, etc; (2) on duty; (3) working year in primary healthcare institutions for ≥ 6 months; (4) communicate without barriers to fill in the questionnaire voluntarily and independently; and (5) informed and voluntary participation in this study. This study was conducted by means of a self-administered anonymous paper questionnaire with oral informed consent. The exclusion criteria were as follows: (1) The unreasonable answer, e.g., too long clinical work year. (2) Short answer time. The time required to answer the questionnaire was not < 10 min (the minimum answer time tested by our research group was 12 min). (3) Inconsistent answers to trap items. Our research group set up two items with the same but different expressions in the questionnaire (trap items). If the answers to the two items were inconsistent, we excluded the questionnaire.

Data were collected in face-to-face interviews by researchers following the principle of full sampling. First, the researchers used unified instructions to explain the purpose and significance of this study to the patients who met the inclusion criteria and asked for their written informed consent. Then, participants were then invited to independently complete the structured questionnaires in a meeting room, which typically took 15–20 min. Upon submission, 2 researchers carefully reviewed each questionnaire to ensure completeness and accuracy. During the investigation, participants could withdraw at any time. Data collection lasted for a week. Finally, 520 valid questionnaires were obtained with an effective response rate of 95.77%.

### 3.2 Variables measurements

#### 3.2.1 Temporal leadership

Temporal leadership was measured using Mohammed and Nadkarni's scale with 7 items that were rated by subordinates (Mohammed and Nadkarni, 2011), such as “My team leader reminds me of important deadlines”, “My team leader effectively coordinates members' work to get the job done”, “My team leader prepares and coordinates for emergencies, difficulties, and emerging issues in a timely manner” and “My team leader prioritizes tasks and allocates time efficiently for each task” etc. The Cronbach's alpha coefficient for this scale in this study was 0.962.

#### 3.2.2 Time management

Time management was measured using Brodowsky et al.'s (2008) Time Management Scale, which consists of 4 items rated by the subordinate, such as “I always want to know how long it will take to complete a task,” “I schedule everything and I plan it in advance,” and “I always want to know how long it will take to complete a task. ” etc. The Cronbach's alpha coefficient of the scale in this study was 0.858.

Work-Family enrichment was measured using Carlson's Work Family enrichment Scale (Carlson et al., 2006). The questionnaire is divided into two parts consisting of 18 items: work-family enrichment and family-work enrichment. This paper only measures work-to-family enrichment in this direction, which consists of 9 items, such as “Helps me to understand different viewpoints and this helps me be a better family member”, “Helps me to gain knowledge and this helps me be a better family member”, “Helps me acquire skills and this helps me be a better family member” etc. In this study, Cronbach's alpha coefficient of the scale was 0.942.

Quiet quitting is based on the quiet quitting Scale developed by Galanis et al. (2023a). There are 9 items such as “I do only the most basic or minimal tasks and nothing more”, “I often pretend to be working to avoid another task” etc. The Cronbach's alpha coefficient of the scale in this study was 0.787.

Organizational communication was measured using the Organizational Communication subscale of Denison's Organizational Culture Scale (Denison, 1990), which consists of three items, such as “I agree that the purpose of communication is clear in the organization”, “I agree that communication is smooth in the organization” and “I agree that communication is effect in the organization”. In this study, the internal consistency coefficient of this scale was 0.912.

All items were responded to a Likert 5 scale, 1 referred to strongly disagree and 5 referred to strongly agree. Higher scores indicate higher level of above variables. Before launching the study, we pre-tested the instrument with 30 PHC workers to identify any problem linked to items, and to identify problems related to comprehension, flow, and duration of questionnaires. The pretest revealed no major problems linked to duration, structure, content, and flow. All questionnaires with the factor loadings of CFA were not < 0.8.

### 3.3 Statistical analysis

Statistical analysis R studio 4.3.2 was used for descriptive statistics, correlation analysis, and Cronbach's alpha reliability estimate. Mplus8.3 was used to test the hypothesized moderated mediation model using the maximum likelihood method of parameter estimation (Cabrera et al., 2024; Preacher and Hayes, 2008). The indirect effect was evaluated by bootstrapping procedures with 5000 bootstrap samples. If the effect confidence interval does not include 0, it is statistically significant. The following indices were used to test the hypothetical model's data fit: the chi-square test (χ^2^), the root mean square error of approximation (RMSEA < 0.08), the Tucker-Lewis index (TLI > 0.90), the Bentler's comparative fit index (CFI > 0.90), normed fit index (NFI >0.90 excellent) and incremental fit index (IFI > 0.90) (Hu and Bentler, 1999; Preacher and Hayes, 2008).

## 4 Result

### 4.1 Demographic characteristics of participants

Table 1 presents demographic characteristics of these 520 medical staff. The female PHC workers accounts for 63.7%. The average age of these PHC workers is around 39.231 ± 10.758, with around 15 average working years. There are 48.4% clinical physicians and nurses, 14.2% medical technicians, 22.8% public health physicians, and 14.6% medical assistants. 91.2% of PHC workers are covered by health insurance and 56.6% of them are staffing of government affiliated institutions.

**Table 1:** Demographics information of medical staff (*n* = 520).

**Demographic**	***N* (%)/M ±SD**
**Gender**	
Male	189 (36.3%)
Female	331 (63.7%)
**Age**	39.231 ± 10.758
**Educational level**	
Junior college and below	77 (14.8%)
University degree	443 (85.2%)
Master degree and above	0 (None)
**Clinical working year**	15.358 ± 11.668
**Occupation**	
Clinical physician and nurse	252 (48.4%)
Medical technician	74 (14.2%)
Public health physician	119 (22.8%)
Support staff	75 (14.6%)
**Work income**	5.763 ± 2.435
**Professional title**	
No title	94 (18.1%)
Primary title	248 (47.7%)
Middle title	150 (28.8%)
Vice-senior title	26 (5%)
Senior title	2 (0.4%)
**Social insurance**	
Yes	474 (91.2%)
No	46 (8.8%)
**Staffing of government affiliated institutions**	
Yes	292 (56.6%)
No	228 (43.4%)

N, number; M, mean; SD, standard deviation.

### 4.2 Common-method deviation test

Harman's one-way test was used to test the collected data for common-method bias. The results extracted 12 factors with characteristic roots greater than one, of which the maximum factor variance explained only 33.53% (< 40%). This indicates no significant common method bias in this study.

### 4.3 Correlation analysis of temporal leadership, time management, work-family enrichment, organizational communication, and quiet quieting

As shown in Table 2, significant correlations were found between temporal leadership, time management, work-family enrichment, organizational communication, and quiet quieting scores. The results indicate that temporal leadership is positively associated with time management (*r* = 0.601, *P* < 0.01), work-family enrichment (*r* = 0.567, *P* < 0.01) and organizational communication (*r* = 0.517, *P* < 0.01). Time management is positively correlated with work-family enrichment (*r* = 0.575, *P* < 0.01) and organizational communication (*r* = 0.454, *P* < 0.01). Work-family enrichment is positively correlated with organizational communication (*r* = 0.414, *P* < 0.01). In addition, we found that quiet quitting is negatively correlated with temporal leadership (*r* = −0.584, *P* < 0.01), time management (*r* = −0.485, *P* < 0.01), Work-family enrichment (*r* = −0.505, *P* < 0.01) and organizational communication (*r* = −0.501, *P* < 0.01).

**Table 2:** Mean scores and correlation analysis of temporal leadership, time management, work-family enrichment, quiet quitting and organizational communication.

**Variables**	**Mean**	**SD**	**Temporal leadership**	**Time management**	**Work-family enrichment**	**Quiet quitting**	**Organizational communication**
Temporal leadership	5.747	0.791	0.903				
Time management	3.326	0.53	0.601^**^	**0.837**			
Work-family enrichment	4.115	0.54	0.567^**^	0.575^**^	**0.829**		
Quiet quitting	2.121	0.601	−0.584^**^	−0.485^**^	−0.505^**^	**0.746**	
Organizational communication	3.988	0.549	0.517^**^	0.454^**^	0.414^**^	−0.501^**^	**0.922**

^**^P < 0.05, values in bold on the diagonal represent the AVE.

### 4.4 The effect of temporal leadership on quiet quitting with chain mediating role of time management and work-family enrichment

**Figure 2:**
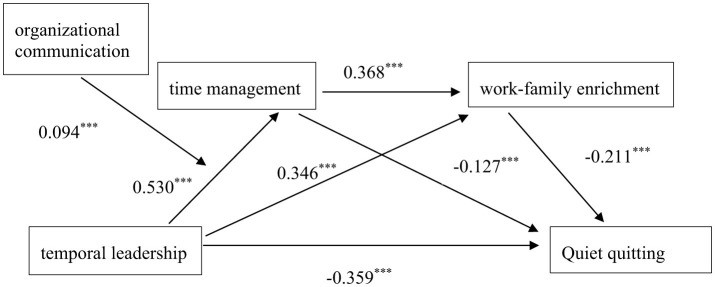
Effects of temporal leadership on quiet quitting among primary healthcare workers: a moderated chain mediation model. All the path coefficients were standardized. ***p < 0.001. Model fit indices: RMSEA = 0.066; TLI = 0.911; CFI = 0.907; NFI = 0.902; IFI = 0.903.

Path coefficients of the moderated mediation model can be seen from Table 3 and Figure 2. The temporal leadership had a negative effect on quiet quitting (β = −0.359, *P* < 0.001), a positive effect on time management (β = 0.530, *P* < 0.001) and work-family enrichment (β = 0.346, *P* < 0.001), separately. Time management and work-family enrichment had negative effects on quiet quitting (β = −0.127, *P* < 0.05; β = −0.211, *P* < 0.05), respectively. And, time management had a positive effect on work-family enrichment (β = 0.368, *P* < 0.001). The bootstrap 95% CI confirms the significant indirect effects of time management [Effect = −0.068, 95% = (−0.134, −0.003)] and work-family enrichment [Effect = −0.073, 95% = (−0.125, −0.021)] in the relationship between temporal leadership and quiet quitting (Table 4). These results indicate that time management and work-family enrichment not only partially mediate the relationship between temporal leadership and quiet quitting, but also have a chain mediating effect on them [Effect = −0.041, 95% = (−0.069, −0.015)].

**Table 3:** Pathway coefficients of the moderated chain mediation model.

**Path**	**β**	**Boot SE**	**95% CI**	***P*-value**
Temporal leadership ->quiet quitting	−0.359	0.070	(−0.494, −0.225)	0
Temporal leadership -> time management	0.530	0.047	(0.446, 0.623)	0
Time management -> quiet quitting	−0.127	0.061	(−0.243, −0.013)	0.038
Temporal leadership -> work-family enrichment	0.346	0.056	(0.241, 0.446)	0
Work-family enrichment -> quiet quitting	−0.211	0.062	(0.328, 0.096)	0.001
Time management -> work-family enrichment	0.368	0.051	(0.263, 0.455)	0
Organizational communication x temporal leadership -> time management	0.094	0.030	(0.032, 0.152)	0.002

β, standardized regression coefficient; SE, standard error; 95% CI, the lower and upper limit of 95% confidence interval.

**Table 4:** Standardized bootstrap estimates and 95% confidence intervals for direct, indirect and total effects by bootstrap methods.

**Relationships**	**Effect**	**Boot SE**	**95% CI**	
			**LLCI**	**ULCI**
Total effect: Total indirect effect + Direct effect	−0.545	0.041	−0.627	−0.463
Direct effect: Temporal leadership -> quiet quitting	−0.361	0.070	−0.501	−0.241
Total indirect effect: Temporal leadership->quiet quitting	−0.185	0.042	−0.269	−0.101
Ind1: Temporal leadership -> time management -> quiet quitting	−0.068	0.033	−0.134	−0.003
Ind2: Temporal leadership -> work-family enrichment -> quiet quitting	−0.073	0.026	−0.125	−0.021
Ind3: Temporal leadership -> time management -> work-family enrichment -> quiet quitting	−0.041	0.014	−0.069	−0.015

SE, standard error; LLCI, the lower limit of 95% confidence interval; ULCI, the upper limit of 95% confidence interval. Ind1, Ind2, and Ind3 is the specific indirect effect of the path. All the path coefficients were standardized.

### 4.5 The moderating effect of organizational communication

**Figure 3:**
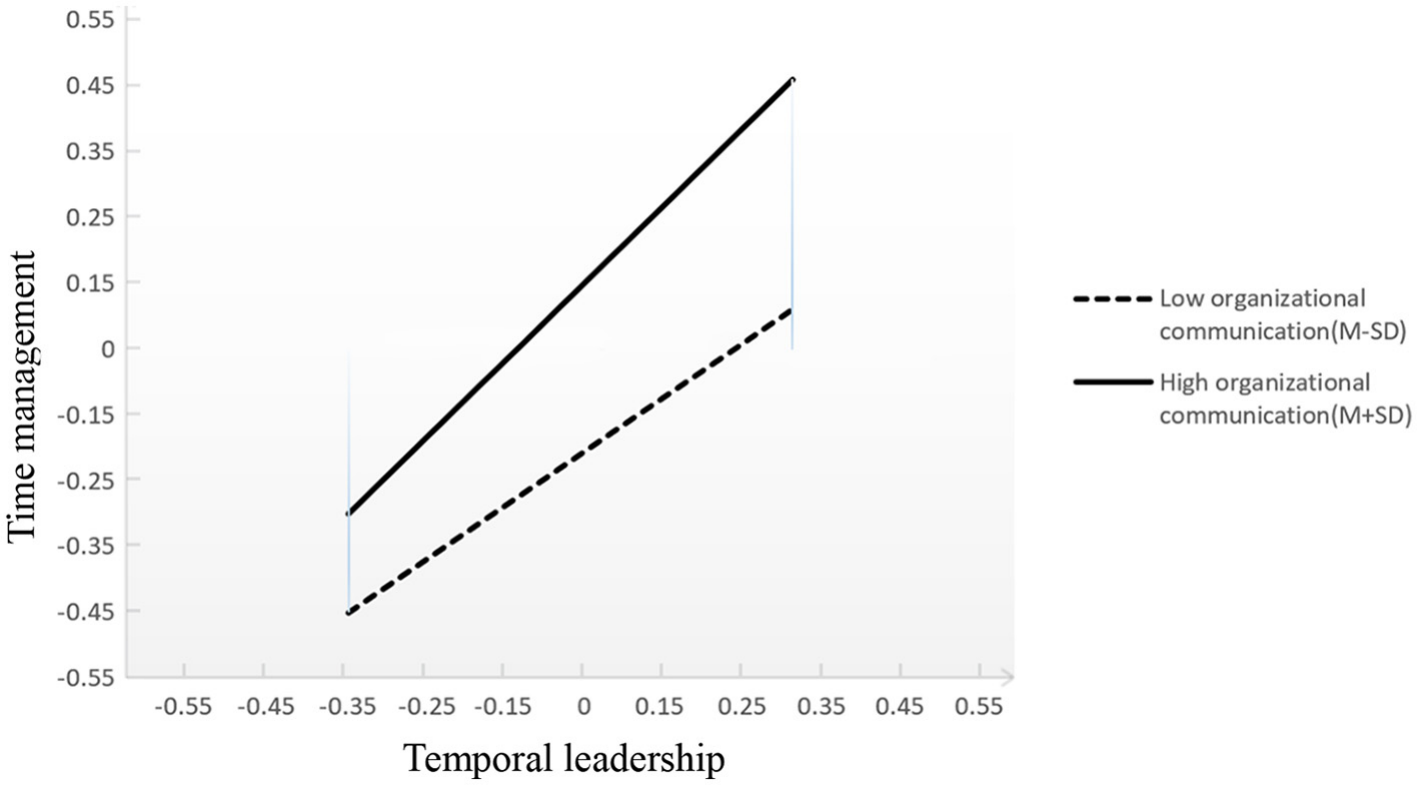
Organizational communication moderates the relationship between temporal leadership and time management. M, mean; SD, standard deviation.

As shown in Table 3 and Figure 3, there was a significant interaction effect between temporal leadership and organizational communication [β = 0.094, SE = 0.03, *P* < 0.01, 95% CI = (0.032, 0.152)] on time management. To provide a better interpretation of this interaction result, the relationship between temporal leadership and time management for high level (mean values minus one standard deviation) and low level of organizational communication (mean values minus one standard deviation) was plotted. The result in Table 5 indicated that the effect of temporal leaderships on individual time management was moderated by organizational communication. The positive predictive effect of temporal leadership on time management was significant in low organizational communication group [Effect = 0.401, 95% CI = (0.289, 0.527)] and then significantly enhanced in the high organizational communication group [Effect = 0.579, 95% CI = (0.472, 0.698)]. The positive predictive effect of temporal leadership on time management was significantly enhanced in high level of organizational communication. In addition, when one of the indirect paths connecting independent and dependent variables was moderated by a variable, the indirect effects of this path were also moderated by this variable (Preacher et al., 2007). Therefore, the indirect effects of temporal leadership on quiet quitting were also moderated by organizational communication (the details are shown in Table 6). Overall, the indirect effects of one indirect pathway were significant for organizational communication: (1) high level of organizational communication: temporal leadership –> time management –> work-family enrichment –> quiet quitting, Effect = −0.032, SE = 0.015, *P* < 0.05, 95% CI = (−0.056, −0.004). (2) low level of organizational communication: temporal leadership –> time management –> work-family enrichment –> quiet quitting, Effect = −0.022, SE = 0.01, *P* < 0.05, 95% CI = (−0.04, −0.003). In contrast, the indirect pathways were not significant for organizational communication: (1) high level of organizational communication: temporal leadership –> time management–> quiet quitting, Effect = −0.029, SE = 0.038, *P* > 0.05, 95% CI = (−0.099, 0.051); (2) low level of organizational communication: temporal leadership–> time management –> quiet quitting, Effect = −0.020, SE = 0.027, *P* > 0.05, 95% CI = (−0.071, 0.036).

**Table 5:** Conditional indirect effect of temporal leadership on time management at values of organizational communication by bootstrap methods.

**Path**	**Level of organizational communication**	**Effect**	**Boot SE**	**95%CI**	
				**LLCI**	**ULCI**
Temporal leadership -> time management	Low (Mean – SD)	0.401	0.061	0.289	0.527
Temporal leadership -> time management	Average (Mean)	0.494	0.052	0.394	0.599
Temporal leadership -> time management	High (Mean + SD)	0.579	0.057	0.472	0.698

LLCI, the lower limit of 95% confidence interval; ULCI, the upper limit of 95% confidence interval; SE, standard error.

**Table 6:** Conditional indirect effect of temporal leadership on quiet quitting at values of organizational communication by bootstrap methods.

**Path**	**Organizational communication**	**Effect**	**Boot SE**	**95%CI**	
				**LLCI**	**ULCI**
Temporal leadership -> time management -> quiet quitting	Low (Mean – SD)	−0.020	0.027	−0.071	0.036
Temporal leadership -> time management-> quiet quitting	Average (Mean)	−0.025	0.032	−0.085	0.043
Temporal leadership -> time management -> quiet quitting	High (Mean + SD)	−0.029	0.038	−0.099	0.051
Temporal leadership -> time management -> work-family enrichment -> quiet quitting	Low (Mean – SD)	−0.022	0.010	−0.04	−0.003
Temporal leadership -> time management -> work-family enrichment -> quiet quitting	Average (Mean)	−0.027	0.012	−0.048	−0.004
Temporal leadership -> time management -> work-family enrichment -> quiet quitting	High (Mean + SD)	−0.032	0.015	−0.056	−0.004

LLCI, the lower limit of 95% confidence interval; ULCI, the upper limit of 95% confidence interval; SE, standard error.

## 5 Discussion

Based on the criterion that a score higher than 2.06 was considered as quiet quitters from Petros's study, the percentage of quiet quitters among PHC workers in our research was about 47%, nearly half of the workforce (Galanis et al., 2023b). It is evident that the working conditions of the medical staff are not promising, and health system executives need to be supplied with appropriate evidence-based strategies to address this critical situation.

First of all, the results revealed an inverse association between temporal leadership and quiet quitting among PHC workers (β = −0.359, *P* < 0.001). This finding is in line with a similar study in area of leadership which reveals that negative influence of transformational leadership on nurses' negative working status like occupational burnout (β = −0.292, *P* < 0.01) (Liu et al., 2019). It is worth noting that both temporal leadership and transformational leadership have similar positive traits, such as high level of accountability and having strong influences on subordinates. Therefore, both of these positive leadership styles can mitigate negative working status of healthcare workers such as burnout or quiet quitting. Comparing to the influencing degree of transformational leadership on nurses' burnout, the results of this study found a higher degree influence of temporal leadership on quiet quitting. This could be because different styles of leadership and negative work status were examined in two studies.

Secondly, this study shows that time management competency, such as an important personality competency, played a significant mediating role in the relationship between temporal leadership and quiet quitting. Our result shows that temporal leadership is the positive predictor of individual time management competency among PHC workers (β = 0.530, *P* < 0.001). Consistent with previous viewpoints that leadership behaviors can influence subordinates' work competency such as improving their innovation efficiency (Ye et al., 2022), our results indicate that leaders with temporal leadership have positive influence on time management competency of employees. Specifically, this is because leaders with temporal leadership typically possess high levels of time management competency themselves, and this trait could motivate subordinators to improve their time management competency (Hoonsopon and Puriwat, 2021). In comparison, earlier study indicates that temporal leadership couldn't directly positively predict the employee's invention competency, but rather through the mediator of vigor (Zhang et al., 2020). This finding contradicts our findings that temporal leadership can directly positively predict time management competency of PHC workers. The reasons may be complex. One possible reason is that two studies explore different professions of employees and their corresponding different employee's competency. Based on authors' knowledge, our study is the first to explore the effect of temporal leaderships on time management competency among PHC workers in health system, which is lack for same study to compare. Moreover, our study shows that the higher time management competency PHC workers have, less likely they are to be quiet quitting (β = −0.127, *P* < 0.001). These results are supported by findings in much more earlier studies which indicate that senior nurses with better time management competency could performance better at work (Amin et al., 2025).

Thirdly, the findings of this study indicate that work-family enrichment mediates the association between temporal leadership and quiet quitting. Specifically, PHC workers' work-family enrichment can be enhanced by temporal leadership, which will in turn mitigate their quiet quitting. A similar finding from a prior leadership research indicates that work-family enrichment is positively influenced by benevolent leadership in bank companies (Wu et al., 2020). The explanations for this are simple. Both temporal leadership and benevolent leadership have their positive leadership traits. In details, temporal leadership can make reasonable work arrangements for employees and benevolent leadership prioritizes the welfare of employees. These positive leadership traits have positive effects on attitudes and behaviors of employees in workplace and family fields, thus finally improve employees' work-family enrichment. And a higher level of work-family enrichment helps to mitigate negative working status such as work-related burnout, quiet quitting and so on (Ollier-Malaterre et al., 2020). Hence, PHC workers with higher work-family enrichment, which can improve individuals' effectiveness and commitment at work and in family, tend to less likely to be quiet quitting (Ollier-Malaterre et al., 2020). In addition, our study indicated that chain mediating role of time management competency and work-family enrichment in the relationship between temporal leadership and quiet quitting. Time management competency can positively predict the work-family enrichment (β = 0.368, *P* < 0.001). Numerous antecedent variables of work-family enrichment, such as work autonomy and work tenure, have been discovered and their potential contributions to work-family enrichment addressed by previous researches (Lapierre et al., 2018). In particular, autonomy can increase work efficiency of employees, which frees up time for family activities and ultimately improves work-family enrichment. Similarly, PHC workers who have higher time management competency are probably going to be more productive and finish their work faster, which will free up more time for family time and lead to greater levels of work-family enrichment.

Finally, our study demonstrates that the relationship between temporal leadership and their time management competency was moderated by organizational communication (β = 0.094, *P* < 0.001). Temporal leadership is more likely to improve employees' time management competencies and mitigate quiet quitting among PHC workers under high level of organizational communication, comparing to those who under low level of organizational communication. This finding is in line with current viewpoints that held that higher levels of organizational communication increase the interactions and influences between superiors and subordinates remote work setting in digital working context currently (Boccoli et al., 2024). Employees and leaders interact more frequently and effectively when there is a high level of organizational communication, and the characteristics of leaders are more likely to influence their subordinates. In details, strong time management competency is typically possessed by leaders with high temporal leadership levels (Chen and Nadkarni, 2017), Based on trait activation theory, when organizational communication is at a higher level, leaders' time management traits are more likely to positively influence their subordinates, thereby enhancing their time management competencies. This aligns with the notion that effective communication within an organization facilitates the activation of leaders' traits, which in turn can improve employees' behaviors and performance. In such contexts, leaders with strong time management skills are better equipped to guide and support their subordinates in developing similar competencies, ultimately benefiting both individual and organizational outcomes. Another quantitative research also reveals that the digital communication skills significantly and positively moderate the relationship between perceived supervisor support and employe's work engagement (β = 0.14, *P* < 0.01) (Boccoli et al., 2024). In comparison to lower influencing degree in our result, the different influencing degree of communication moderator is due to different leadership styles or behavior and studying subjects. The research findings indicate that, under the moderating effect of organizational communication, the mediating effect of time management on the relationship between time leadership (TL) and quiet quitting (QQ) is not significant.

However, under the moderating effect of organizational communication, there is a significant conditional mediating effect between time leadership and quiet quitting. These phenomena can be explained by the work-family conflict theory (Carlson et al., 2000; Dunn, 2025; Greenhaus and Powell, 2006) and the resource conservation theory (Boley, 2025; Mehta and Zhu, 2016). Although organizational communication can enhance the impact of time leadership on the time management capabilities of primary healthcare (PHC) workers, the improved time management competency may not directly alleviate quiet quitting. Resource conservation theory (Boley, 2025) suggests that individuals strive to preserve and accumulate resources (such as time, energy, and property) to cope with life stress. When employees perceive the depletion of these resources, it may lead to occupational burnout and work disengagement. Although organizational communication may improve time management through time leadership, it fails to directly address the depletion of emotional and psychological resources, thereby unable to effectively reduce employees' quiet quitting. According to the work-family enrichment theory (Dunn, 2025), positive leadership styles, good work support environments, and personal work autonomy are all inducing resources for work-family enrichment, which help reduce turnover intentions and promote work performance. While organizational communication can enhance employees' work competency, if it fails to achieve resource enrichment in both work and family domains, it may not directly reduce quiet quitting. When both work and family domains lack sufficient resources, PHC workers with strong time management skills have to allocate more time reduced from work to the family domain, thereby creating a work-family enrichment. Under the influence of organizational communication, these inducing resources can promote work-family balance, enhance employee well-being, and finally reduce the risk of quiet quitting. Therefore, the chained mediating effect incorporating the work-family enrichment effect as an additional mediating variable is of significant importance. This effect can alleviate stress, provide emotional support, and enhance employees' work motivation, ultimately reducing quiet quitting. Therefore, while organizational communication can enhance time management competencies, if this enrichment fails to bring about resource gains from work to family (i.e., simultaneously meeting task management and family needs), time management competencies enhanced solely through organizational communication may be insufficient to effectively mitigate quiet quitting.

## 6 Conclusion

Based on authors' knowledge, this study makes a significant contribution to the literature by being the first to explore how temporal leadership can mitigate quiet quitting among PHC workers, which contributes to revealing the influencing mechanism of temporal leadership on quiet quitting. In summary, temporal leadership not only has a directly positive effect on mitigating quiet quitting among PHC workers, but can also indirectly mitigate quiet quitting through time management and work-family enrichment of PHC workers. The degree of quiet quitting among PHC employees is less severe when leaders exhibit a higher level of temporal leadership, and they are also more likely to exhibit higher levels of time management competency and work-family enrichment. In addition, the relationship between temporal leadership and time management is moderated by organizational communication. Specifically, the enhancement effect of temporal leadership for leaders on time management competency of PHC workers would be increased under high level of organizational communication when compared with that under low level of organizational communication. These findings contribute to the literature on leadership's role in employee quitting-related behavior and provide valuable insights for preventing workforce decline in healthcare settings.

This study has significant practical implications, particularly for low-resource healthcare environments where staff shortages, heavy workloads, and limited managerial capacity often exacerbate employee disengagement. In such contexts, quiet quitting can be especially harmful, not only diminishing service efficiency but also jeopardizing the continuity and quality of patient care. Our findings suggest that adopting temporal leadership practices, such as rational scheduling, clear time expectations, and coordinated workflows, provides a cost-effective means of mitigating quiet quitting without requiring major structural changes. Additionally, by strengthening time management competencies and promoting work-family enrichment, managers can improve employee resilience and motivation, even in resource-constrained settings. Transparent and supportive organizational communication can further enhance these positive outcomes. These strategies are particularly valuable for primary healthcare systems striving to maintain workforce stability and ensure service delivery despite budgetary and operational constraints.

## 7 Limitation and future research directions

This study has the following limitations. Firstly, the cross-sectional study limited its ability to confirm a causal relationship between temporal leadership, time management, work-family enrichment and quiet quitting. Therefore, it would be better to conduct follow-up study for higher level evidence. Secondly, data in this study came from the self-assessment questionnaires of the primary healthcare workers, many factors could have potentially affected the results of self-assessment questionnaires, including self-defensiveness, pretending, personal emotion, and other attitudes. The follow-up study may be combined with other assessments or observation methods to improve the reliability of the study. Thirdly, the sample for this study was drawn from all primary healthcare institutions in a demonstrate county for poverty eradication in China, and it is unclear whether the findings can be generalized to other populations. Finally, as digital transformation continues to empower management practices, the potential role of digital tools in enhancing leadership effectiveness and organizational communication will likely emerge as an important area for future research.

## Data Availability

Publicly available datasets were analyzed in this study. This data can be found here: the data sets generated and/or analyzed during this study are available from the corresponding author on reasonable request.
