# Supplementary material for: The effect of temporal leadership on quiet quitting among primary healthcare workers: the chain-mediating role of time management competency and work-family enrichment and moderating role of organizational communication
Source: Front Psychol. 2025 Sep 3;16:1616354. doi: 10.3389/fpsyg.2025.1616354 (PMC12442560; doi:10.3389/fpsyg.2025.1616354)
Supplement: Supplementary file 1 [file Data_Sheet_1.docx]

Supplementary Material

**APPENDIX A：The dimensions and measurement items of temporal leadership**

| Dimensions | Items |
| --- | --- |
| temporal leadership | TL1: My project leader reminds members of important deadlines |
|  | TL2: My project leader prioritizes tasks and allocate time to each task |
|  | TL3: My project leader prepares and build in time for contingencies, problems, and emerging issues |
|  | TL4: My project leader pace the team so that work is finished on time |
|  | TL5: My project leader urges members to finish subtasks on time |
|  | TL6: My project leader set milestones to measure progress on the project |
|  | TL7: My project leader is effective in coordinating the team to meet client deadlines |

**APPENDIX B：The dimensions and measurement items of time management:**

| Dimensions | Items |
| --- | --- |
| time management | TM1: I always like to know how long a task will take  TM2: I always schedule and pre-plan everything  TM3: I want to know the ending and beginning time  TM4: Managing time is key to success |

**APPENDIX C：The dimensions and measurement items of work-family enrichment:**

| Dimensions | Items |
| --- | --- |
| Work to family development | WD1: Helps me to understand different viewpoints and this helps me be a better family member  WD2: Helps me to gain knowledge and this helps me be a better family member  WD3: Helps me acquire skills and this helps me be a better family member |
| Work to family affect | WA1: Puts me in a good mood and this helps me be a better family member  WA2: Makes me feel happy and this helps me be a better family member  WA3: Makes me cheerful and this helps me be a better family member |
| Work to family capital | WC1: Helps me feel personally fulfilled and this helps me be a better family member  WC2: Provides me with a sense of accomplishment and this helps me be a better family member  WC3: Provides me with a sense of success and this helps me be a better family member |

**APPENDIX D：The dimensions and measurement items of quiet quitting:**

| Dimensions | Items |
| --- | --- |
| Detachment | DE1: I do the basic or minimum amount of work without going above and beyond.  DE2: If a colleague can do some of my work, then I let him/her do it.  DE3: I take as many breaks as I can.  DE4: I often pretend to be working in order to avoid another task. |
| Lack of initiative | LI1: I don’t express opinions and ideas about my work because I am afraid that the manager assigns me more tasks.  LI2: I don’t express opinions and ideas about my work because I think that working conditions are not going to change.  LI3: I often take initiative at your work. (R) |
| Lack of motivation | LM1: I find motives in my job. (R)  LM2: I feel inspired when I work. (R) |

**APPENDIX E：The dimensions and measurement items of organizational communication:**

| Dimension | Items | Sources |
| --- | --- | --- |
| organizational communication | OCO1: Generally, the purpose of organizational communication is clear  OCO2: Communication within the organization is smooth and effective  OCO3: Mistakes or wasted resources are often caused by poor communication in the workplace (R) | [31] |
